# Longitudinal evaluation of the early auditory gamma-band response and its modulation by attention in first-episode psychosis

**DOI:** 10.1017/S0033291724003052

**Published:** 2024-11

**Authors:** Alfredo L. Sklar, Sayna Matinrazm, Annika Esseku, Fran López-Caballero, Mark Curtis, Dylan Seebold, Natasha Torrence, Vanessa Fishel, Brian A. Coffman, Dean F. Salisbury

**Affiliations:** 1 Department of Psychiatry Pittsburgh, University of Pittsburgh School of Medicine, Pittsburgh, PA, USA; 2 College of Health Professions, Rosalind Franklin University of Medicine and Science, North Chicago, IL, USA

**Keywords:** early auditory gamma-band response, first-episode psychosis, longitudinal analysis, magnetoencephalography, negative symptoms, selective attention

## Abstract

**Background:**

Executive control over low-level information processing is impaired proximal to psychosis onset with evidence of recovery over the first year of illness. However, previous studies demonstrating diminished perceptual modulation via attention are complicated by simultaneously impaired perceptual responses. The present study examined the early auditory gamma-band response (EAGBR), a marker of early cortical processing that appears preserved in first-episode psychosis (FEP), and its modulation by attention in a longitudinal FEP sample.

**Methods:**

Magnetoencephalography was recorded from 25 FEP and 32 healthy controls (HC) during active and passive listening conditions in an auditory oddball task at baseline and follow-up (4–12 months) sessions. EAGBR inter-trial phase coherence (ITPC) and evoked power were measured from responses to standard tones. Symptoms were assessed using the Positive and Negative Syndrome Scale (PANSS).

**Results:**

There was no group difference in EAGBR power or ITPC. While EAGBR ITPC increased with attention in HC, this modulation was impaired among FEP. Diminished EAGBR modulation in FEP persisted at longitudinal follow-up. However, among FEP, recovery of EAGBR modulation was associated with reduced PANSS negative scores.

**Conclusion:**

FEP exhibit impaired executive control over the flow of information at the earliest stages of sensory processing within auditory cortex. In contrast to previous work, this deficit was observed despite an intact measure of sensory processing, mitigating potential confounds. Recovery of sensory gain modulation over time was associated with reductions in negative symptoms, highlighting a source of potential resiliency against some of the most debilitating and treatment refractory symptoms in early psychosis.

## Introduction

Schizophrenia is a complex disorder characterized by multiplex symptoms and profound functional deficits. Dysfunctions in executive brain networks (Minzenberg, Laird, Thelen, Carter, & Glahn, 2009) and neurocognitive functions (Green, Kern, Braff, & Mintz, 2000) are contributors to disease morbidity, and there now exists considerable evidence for disruptions of more fundamental sensory-perceptual processes (Javitt, 2009; Salisbury, Collins, & McCarley, 2010; Sklar, Coffman, & Salisbury, 2020). However, interactions between these systems in psychosis, particularly mechanisms guiding executive control over early perceptual processing, remain understudied. Modulation of neural responses to basic stimuli is critical to promoting organized, goal-directed thoughts and behaviors by enhancing processing of more salient aspects of the environment and suppressing less relevant ones. As such, examining the influence of attention networks on processing within sensory cortices in psychosis, a condition characterized by dysfunctions of neural connectivity (Jensen et al., 2024; Phalen, Coffman, Ghuman, Sejdić, & Salisbury, 2020; Sklar, Coffman, Longenecker, Curtis, & Salisbury, 2022), is vital to the characterization of primary sources of disease debility.

Within the auditory system, previous work (Rosburg, Boutros, & Ford, 2008) demonstrated impaired enhancement of the N100 auditory evoked potential, a putative marker of sensory integration (Näätänen & Picton, 1987), by attention in schizophrenia. Our lab recently replicated this finding in a first-episode psychosis (FEP) sample (Ren, Fribance, Coffman, & Salisbury, 2021) and found reduced N100 enhancement to be associated with worse neurocognitive performance, negative symptoms, and social and occupational functioning. As an extension to this work, we examined cortical sources of M100-related activity utilizing magnetoencephalography (MEG) recordings during an oddball task and observed both reductions of overall auditory cortex (AC) activity and diminished enhancement of this response by attention (Curtis, Ren, Coffman, & Salisbury, 2023a). Follow-up analyses revealed this impaired modulation was associated with reduced connectivity between auditory cortices and a broad attention network in the patient sample providing a potential mechanism underlying executive control, or lack thereof, over sensory-perceptual processing in early psychosis (Curtis, Sklar, Coffman, & Salisbury, 2023b).

While N/M100 indices provide valuable information regarding auditory cortical functioning, they capture relatively later processing stages of perception ~100 ms post-stimulus compared to initial activity within primary AC occurring at ~20 ms. In contrast, the early auditory gamma-band response (EAGBR), a high-frequency response evoked about 50 ms following presentation of discrete auditory stimuli, reflects initial stages of sensory-level stimulus registration within the cortex. Unlike the N/M100, the EAGBR does not appear to be diminished in FEP (Oribe et al., 2019; Sklar et al., 2024). In fact, a larger EAGBR appears to be associated with more severe symptoms in this cohort (Sklar et al., 2024; Taylor, McCarley, & Salisbury, 2013). Highlighting the influence of executive control over the earliest stages of information processing within the cortex, the EAGBR does appear to be modulated by attention in healthy adults (Debener, Herrmann, Kranczioch, Gembris, & Engel, 2003; Tiitinen, May, & Näätänen, 1997). However, attentional modulation of the EAGBR and its relationship to disease burden remains unstudied in early psychosis.

Beyond decisions regarding the indices of cortical processing to examine, consideration of disease stage is critical to the investigation of sensory-perceptual processing and its modulation by attention. For example, while studies suggest that the EAGBR may be intact at disease onset, a recent longitudinal analysis found deficits in the EAGBR emerging over a 1-year follow-up period (Oribe et al., 2019) and chronicity of the illness may account for some of the discrepancies within the EAGBR literature. Longitudinal changes of the M100-related auditory response have also been observed within the first year of a psychotic illness including recovery of its modulation by attention (Coffman, Curtis, Sklar, Seebold, & Salisbury, 2023). Importantly, increased sensitivity of M100-related cortical activity to the effects of attention was significantly associated with improvements in symptoms over the follow-up period. These findings highlight the dynamic nature of the illness during its first year as well as the utility of markers for early sensory-perceptual auditory processing in tracking disease progression during this sensitive period.

In the present study, we have leveraged the exquisite spatio-temporal resolution of merged MEG and magnetic resonance imaging (MRI) data to examine longitudinal changes in AC EAGBR modulation by attention and its relationship to disease burden in FEP. MEG was recorded during an auditory oddball paradigm at baseline and again 4–12 months later. Symptoms and community functioning were assessed at both timepoints and correlations between EAGBR measures, clinical assessments, and their change over time were examined. In-line with previous findings, we hypothesized a preserved EAGBR among FEP at baseline testing, with a larger response associated with greater disease burden. Similar to the N/M100 response, we also predicted impaired attention modulation of the EAGBR at baseline among FEP that recovers over time.

## Methods

### Participants

Data were collected from 51 healthy controls (HC) and 42 FEP at baseline. Among these, 38 (74.5%) HC and 26 (61.9%) FEP returned for follow-up. Data from six HC and one FEP were excluded from further analysis to ensure groups were matched on age, sex-distribution, parental socioeconomic status, and pre-morbid IQ, resulting in a final longitudinal sample of 32 HC and 25 FEP. Follow-up testing sessions occurred 4–12 months following baseline with no significant difference in interscan interval between the groups (HC: 190 ± 56 days; FEP: 218 ± 95 days; *p* = 0.17). The current longitudinal sample largely overlapped with a previous study (Coffman et al., 2023) examining attentional modulation of the M100-related AC response (50/57 participants) and partially overlapped with a previous study (Sklar et al., 2024) examining modulation of the baseline EAGBR by stimulus intensity (42/57 participants).

Assessments for all participants included the Structured Clinical Interview for DSM-IV (SCID-IV), current scores on the Global Functioning: Role and Social (GF:Role/Social) scales, the MATRICS Consensus Cognitive Battery (MCCB), and the Wechsler Abbreviated Scale of Intelligence (WASI). MCCB data were not collected for three participants (two HC; one FEP) at follow-up. Participants were excluded if they had a history of concussion or head injury with sequelae, neurological comorbidity, history of substance dependence, a positive urine drug screen on day of testing aside from cannabis, <9 years of education, or abnormal hearing as assessed by audiometry (within 30 dB nHL and <15 dB difference between ears from 500 to 4000 Hz).

FEP were recruited from Western Psychiatric Hospital inpatient and outpatient services. Diagnoses, determined by SCID-IV, included: 20 schizophrenia, 2 psychosis not otherwise specified, 1 major depressive disorder with psychotic features, and 2 bipolar disorder with psychotic features. FEP were enrolled within 1 year of first clinical contact for a psychotic symptom and had less than 1 year of total antipsychotic exposure. Three FEP were unmedicated at baseline and six at follow-up. Disease symptoms were assessed using the Positive and Negative Syndrome Scale (PANSS).

### Auditory paradigm

Participants completed two blocks of an auditory oddball task typically used to assess attentional gain of early sensory-perceptual neurophysiological markers including the EAGBR (Gurtubay, Alegre, Labarga, Malanda, & Artieda, 2004). In one block, participants were instructed to ignore the tones being played while they attended to a silent video. In the second, they were instructed to ignore the video and attend to the tones, responding to deviant tones via button-press which provided a behavioral measure to assess engagement with the task. Each block consisted of 340 standard (1000 Hz, 50 ms duration, 10 ms rise/fall) and 60 deviant (1200 Hz, 50 ms duration, 10 ms rise/fall) tones with a stimulus onset asynchrony of 1050–1550 ms. Block order was counter-balanced between participants.

### MRI acquisition and processing

Structural MRI scans were obtained for each participant. A Siemens Magnetom Prisma Fit 3T system was used to acquire *T*1 (TR/TE/TI = 2400/2.22/1000 ms, flip angle = 7°, FOV = 256 × 240 mm^2^, voxel size = 0.8 mm^3^, 208 slices, GRAPPA acceleration factor = 2) and *T*2 (TR = 3200 ms, TE = 563 ms, FOV = 256 × 240 mm^2^, voxel size = 0.8 mm^3^, 208 slices) weighted images as well as a 10 min resting-state functional MRI scan (TR = 800 ms, TE = 37 ms, multiband factor = 8, flip angle = 52°, FOV = 208 × 208 mm^2^, voxel size = 2.0 mm^3^, 72 slices). MRI scans were processed according to Human Connectome Project (HCP) pipelines (Glasser et al., 2013) to obtain HCP cortical parcellations of the cortical surface for each participant using multivariate mapping from resting-state networks, sulcal/gyral markers, and *T*1/*T*2 ratios. For a detailed description for the application of this processing pipeline, see Curtis et al. (2023a).

### MEG acquisition and processing

MEG data were recorded using a 306-channel (128 triplets: 1 magnetometer and 2 planar gradiometers) Elekta-Neuromag Vectorview system. Data were sampled at 1000 Hz with an online bandpass filter of 0.1–330 Hz. Bipolar leads were placed at the outer canthi of both eyes to monitor horizontal eye movements and single-channel leads were placed below the left eye and on the left clavicle to monitor blinks and electrocardiogram, respectively. Four head-positioning indicator (HPI) coils were placed on the scalp to monitor head movements throughout the scan and a 3D digitizer (ISOTRAK; Polhemus, Inc., Colchester, VT) was used to record the location of the HPI coils relative to the scalp for later source reconstruction.

Initial pre-processing steps were conducted to remove artifacts from the data. Neuromag MaxFilter software (http://imaging.mrc-cbu.cam.ac.uk/meg/Maxfilter_V2.2) was used to correct for head motion and apply the temporal extension of the signal space separation (Taulu & Hari, 2009). Data were then imported into EEGLAB (Delorme & Makeig, 2004). A 0.5 Hz high-pass filter was applied, bad channels and corrupted segments of data were identified, and an adaptive mixture independent component analysis was used to isolate and remove eye movement, blink, and cardiac artifacts.

Additional processing steps to isolate power and inter-trial phase coherence (ITPC) of the EAGBR and localize its cortical sources were conducted in Brainstorm (Tadel, Baillet, Mosher, Pantazis, & Leahy, 2011). Low-pass (100 Hz) and notch (60 Hz) filters were applied to the data. While both standard and deviant tones elicit modulation of the EAGBR utilizing the current auditory oddball paradigm (Gurtubay et al., 2004), analyses based on standard tone stimuli provide a more reliable measure of the EAGBR given the significantly enhanced signal-to-noise ratio (Hall et al., 2011; Taylor et al., 2013). As such, data from standard tone trials were segmented (−300 to 600 ms) and baseline-corrected (−150 to 0 ms). Trials exceeding ±5 pT were rejected. MEG sensor data were registered to each participant's MRI scan. A forward solution was modeled as overlapping spheres based on sensor locations and a noise covariance matrix was calculated from the baseline window of all trials. Source activity was constrained to the cortical surface and estimated using the minimum norm estimation with an orientation constraint of 0.2 and depth-weighting applied. Dynamic statistical parametric mapping values were calculated at each vertex (1500 per hemisphere) normalizing the current estimate to the pre-stimulus baseline covariance. The AC region of interests included primary auditory, lateral belt, and parabelt parcels from the HCPMMP atlas for each hemisphere.

**Figure 1.**
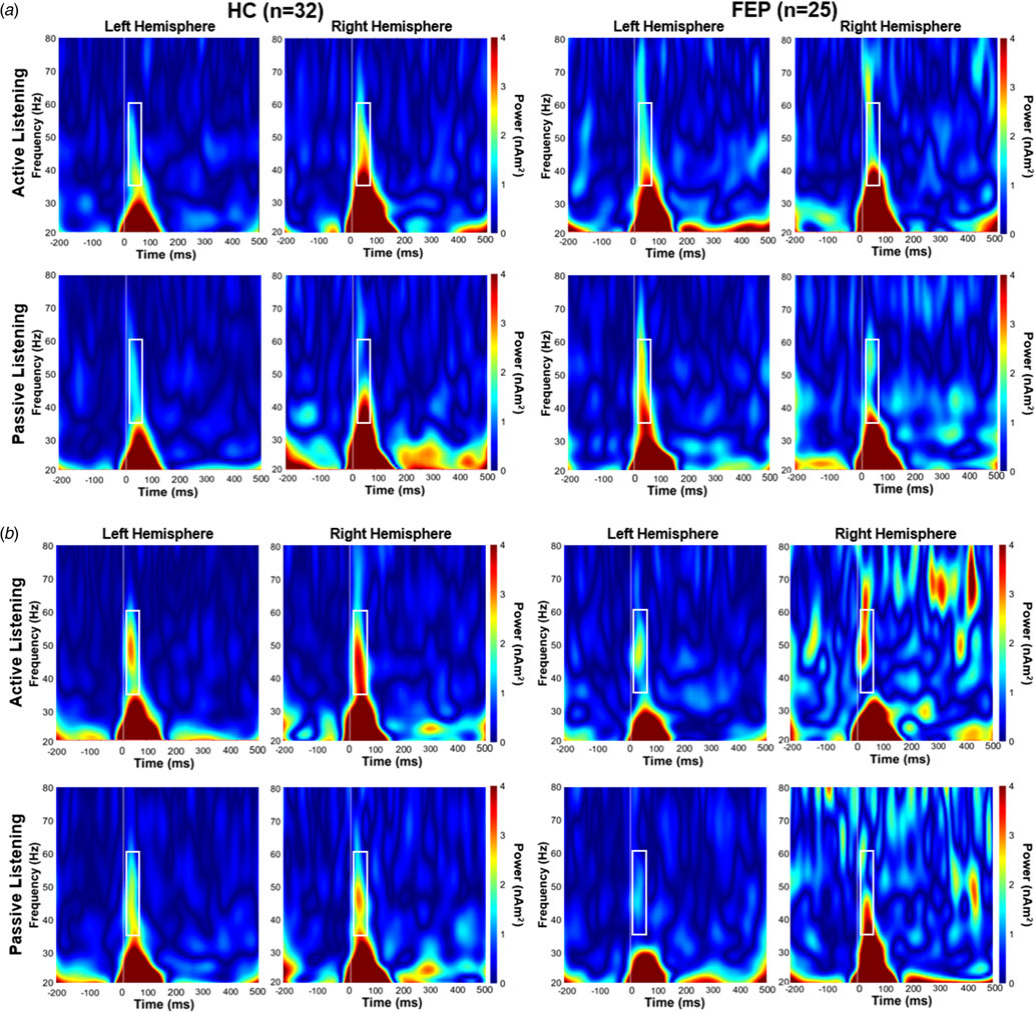
Spectrograms of EAGBR power elicited by tones during active and passive listening conditions for baseline (a) and follow-up (b) timepoints. White box indicates time (20–60 ms) and frequency (35–60 Hz) window for calculation of the EAGBR.

**Figure 2.**
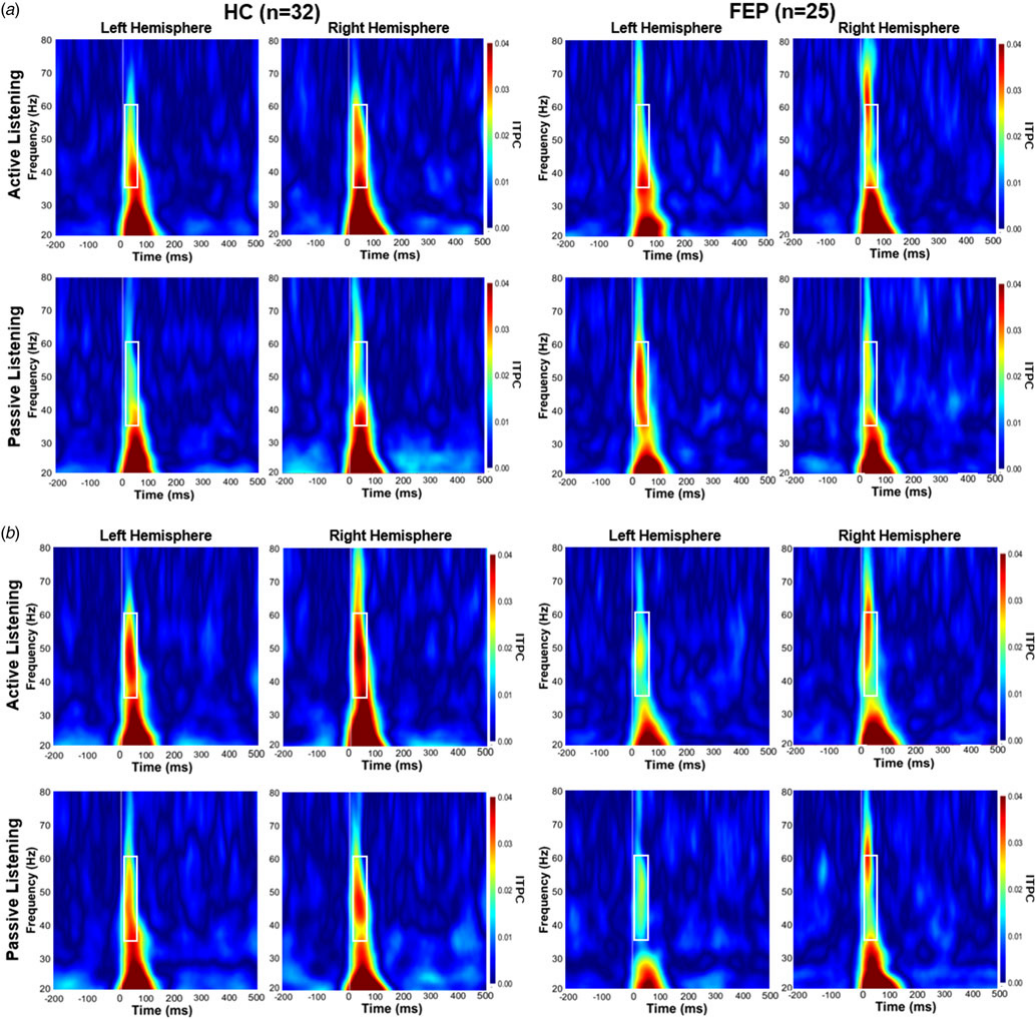
Spectrograms of EAGBR ITPC elicited by tones during active and passive listening conditions for baseline (a) and follow-up (b) timepoints. White box indicates time (20–60 ms) and frequency (35–60 Hz) window for calculation of the EAGBR.

Time–frequency analysis was conducted on source-localized activity data within the AC to assess ITPC and power of the EAGBR. A Morlet wavelet transform (*f* = 1 Hz; full-width at half maximum = 3 s) was applied at 1 Hz steps from 20 to 80 Hz to individual trials and trial-averaged data. Evoked power was extracted from the trial-averaged sources. ITPC, a measure of phase variability relative to a stimulus with values ranging from 0 (no coherence) to 1 (complete coherence), was assessed using phase data from signals recorded during each individual trial. EAGBR power and ITPC calculated at each vertex was averaged across the AC, and baseline (−150 to 0 ms) corrected. Time (20–60 ms) and frequency (35–60 Hz) windows used to calculate the EAGBR were based on previous work using the same processing pipeline (Sklar et al., 2024) and goodness-of-fit with observed time–frequency plots (Figs 1 and 2).

### Data analysis

Demographic variables were compared between the groups using χ^2^ and *t* tests (Table 1). Task performance, GF:R/GF:S, and MCCB were compared between the groups over time using a 2 (group: HC/FESz) by 2 (time: baseline/6 m) repeated-measures analysis of variance (ANOVA), and clinical assessments for FEP were compared between timepoints using paired *t* tests (Table 2). EAGBR power and ITPC were compared between the groups across timepoints using a 2 (group: HC/FESz) by 2 (time: baseline/follow-up) by 2 (attention: attend/ignore tones) by 2 (hemisphere: LH/RH) repeated-measures ANOVA. Simple effects and pairwise comparisons were examined using repeated-measures ANOVA or *t* tests. To examine potential selection bias created by our longitudinal assessment, baseline demographic, clinical, and neurophysiological endpoints were examined between participants that did and did not return for follow-up testing. Effects examined using ANOVA and *t* tests were considered significant for *p* values <0.05 and trend-level for *p* values <0.1. Given our primary interest in the impact of executive control over sensory cortical processing, associations between EAGBR modulation (EAGBR power/ITPC during active minus passive listening conditions) and clinical assessments at each timepoint as well as between the change in each over time were assessed using Spearman's rank-order correlations. As a secondary analysis, correlations were also conducted between EAGBR values in each condition and clinical assessments. Correlations were subjected to false-discovery rate correction for multiple comparisons and considered significant at *q* < 0.1 (Benjamini & Yekutieli, 2005).

**Table 1.** Group demographic data (mean ± s.d.)

	HC (*n* = 32)	FEP (*n* = 25)	*t*/χ^2^	*p*
Female/male	6/26	7/18	0.68	0.41
Education (years)	15.56 ± 3.1	13.20 ± 1.7	3.45	0.001
Age (years at baseline)	24.442 ± 5.9	23.80 ± 5.0	0.43	0.67
Race/ethnicity			11.76	0.04
White	22	8		
Black	3	10		
Asian	3	4		
Hispanic	1	1		
Pacific Islander/Native American	3	1		
Mixed race/non-specified	0	1		
Verbal IQ^a^	52.28 ± 5.9	49.36 ± 8.9	1.49	0.14
SES	40.25 ± 13.7	30.00 ± 12.8	2.88	0.006
PSES	46.98 ± 12.6	44.10 ± 11.9	0.88	0.38
Handedness (right/ambidextrous/left)	30/0/2	20/2/3	3.39	0.18
DUP (years)	–	1.42 ± 1.4		
Median DUP (years)	–	1.03		
First clinical contact to baseline scan (weeks)	–	37.42 ± 78.1		
Median first clinical contact to baseline scan (weeks)	–	7.21		

SES, socioeconomic status; PSES, parental socioeconomic status; DUP, Duration of untreated psychosis.

a Vocabulary subtest of the Wechsler Abbreviated Scale of Intelligence.

**Table 2.** Group clinical assessment and EAGBR data across timepoints (mean ± SD)

	HC (*n* = 32)		FESz (*n* = 25)	
	Baseline	Follow-up	Baseline	Follow-up
MCCB-Total	52.72 ± 6.6	55.63 ± 7.2	32.56 ± 14.8	34.32 ± 12.7
GF: Role	9.04 ± 0.1	9.06 ± 0.2	4.76 ± 2.1	5.80 ± 2.3
GF: Social	9.03 ± 0.1	9.09 ± 0.2	4.88 ± 2.1	5.44 ± 2.0
UDS (± for THC)	2/30	5/20	3/29	4/21
PANSS positive	–	–	49.92 ± 11.2	42.12 ± 9.2
PANSS negative	–	–	43.64 ± 9.8	40.72 ± 8.1
Medicated	–	–	22/25	19/25
Dose (CPZeq)			240.7 ± 147.3	270 ± 153.9
Hit rate	0.85 ± 0.2	0.83 ± 0.2	0.76 ± 0.2	0.83 ± 0.2
False-alarm rate	0.01 ± 0.01	0.01 ± 0.01	0.01 ± 0.02	0.01 ± 0.01
EAGBR power				
LH active	1.38 ± 1.9	2.12 ± 2.7	1.91 ± 2.5	1.42 ± 2.5
LH passive	1.24 ± 2.0	1.84 ± 3.7	2.45 ± 3.4	0.84 ± 1.5
RH active	2.51 ± 3.0	2.95 ± 4.7	2.11 ± 2.4	1.76 ± 2.6
RH passive	1.95 ± 3.4	2.14 ± 3.1	1.68 ± 2.2	1.91 ± 4.8
EAGBR ITPC				
LH active	0.024 ± 0.02	0.030 ± 0.03	0.024 ± 0.03	0.019 ± 0.02
LH passive	0.019 ± 0.02	0.026 ± 0.02	0.029 ± 0.03	0.017 ± 0.02
RH active	0.029 ± 0.02	0.036 ± 0.04	0.024 ± 0.02	0.024 ± 0.03
RH passive	0.023 ± 0.02	0.027 ± 0.03	0.021 ± 0.02	0.021 ± 0.03

MCCB, MATRICS Consensus Cognitive Battery; GF: Role, Global Functioning: Role scale; GF: Social, Global Functioning: Social scale; UDS, urine drug screen; PANSS positive, positive symptom component of the PANSS (*t* scores); PANSS negative, negative symptom component of the PANSS (*t* scores); CPZeq, chlorpromazine equivalent dose.

## Results

### Demographics, clinical assessments, and task performance

Full statistical reporting of demographic data is presented in Table 1. Consistent with disease burden on functioning, FEP had fewer years of education (*t*_55_ = 3.45, *p* = 0.001, *d* = 0.92) and lower socioeconomic status (*t*_55_ = 2.88, *p* = 0.006, *d* = 0.77) relative to HC. Groups did not differ in sex distribution, age, WASI vocabulary subscale scores, or parental socioeconomic status (*p*'s > 0.1). Proportion of urine drug screen positive for cannabis, displayed in Table 2, did not differ between the groups at either timepoint (*p*'s > 0.1).

Clinical assessment statistics are present in Table 2. Overall, FEP had lower GF:R (*F*_1,55_ = 105.88, *p* < 0.001) and GF:S (*F*_1,55_ = 131.38, *p* < 0.001) scores relative to HC. There was also a differential effect of time on both GF:R (*F*_1,55_ = 15.08, *p* < 0.001) and GF:S (*F*_1,55_ = 4.06, *p* = 0.049) scores between the groups. While HC showed no change in GF:R over time (*p* = 0.57), FEP exhibited significant increases (*t*_24_ = 3.50, *p* = 0.002, *d* = 0.70) indicating improved role/occupational functioning at follow-up. Regarding GF:S scores, HC showed a small, but significant improvement over time (*t*_24_ = 2.10, *p* = 0.04, *d* = 0.37) while FEP exhibited a relatively larger, trend-level increase (*t*_24_ = 2.02, *p* = 0.06, *d* = 0.41) indicating improved inter-personal function at follow-up. FEP had lower MCCB compared to HC (*F*_1,52_ = 58.74, *p* < 0.001). These scores improved over time across groups (*F*_1,52_ = 8.23, *p* = 0.006), and this effect did not differ between the groups (*p* = 0.66). PANSS positive (*t*_24_ = 4.17, *p* < 0.001, *d* = 8.3) and negative (*t*_24_ = 2.1, *p* = 0.046, *d* = 0.42) symptom subscale scores improved over time.

Task performance data are presented in Table 2. Hit rates did not differ between the groups (*p* = 0.35) or across timepoints (*p* = 0.24). An interaction between group and timepoint was present (*F*_1,55_ = 5.44, *p* = 0.02), with FEP exhibiting a trend toward increased hit rates with time (*t*_24_ = 1.83, *p* = 0.08, *d* = 0.37) and no difference observed among HC (*p* = 0.24). No effect of group, timepoint, or their interaction was observed on false-alarm rates (*p*'s > 0.3).

### EAGBR power

Spectrograms depicting AC EAGBR power for both groups across timepoints are presented in Fig. 1. There were no main effects of group (*p* = 0.55), timepoint (*p* = 0.92), attention condition (*p* = 0.13), or hemisphere (*p* = 0.22) on EAGBR power. A trend-level interaction on EAGBR power was observed between group and timepoint (*F*_1,55_ = 2.79, *p* = 0.098) with HC exhibiting a non-significant increase (baseline: 17.76 ± 16.6; follow-up: 22.68 ± 23.8) and FEP exhibiting a non-significant decrease (baseline: 20.41 ± 16.2; follow-up: 14.88 ± 21.2) over time. No additional interactions were observed (*p*'s > 0.1).

### Early auditory gamma-band response inter-trial phase coherence

Spectrograms depicting AC EAGBR ITPC for both groups across timepoints are presented in Fig. 2. No effect of group (*p* = 0.26), timepoint (*p* = 0.55), or hemisphere (*p* = 0.51) was present. The attend condition produced larger ITPC relative to the ignore condition (*F*_1,55_ = 7.35, *p* = 0.009). Of primary importance, this attention effect differed between the groups (*F*_1,55_ = 4.36, *p* = 0.04). While ITPC did not differ significantly between the groups within either condition (*p*'s > 0.1), HC exhibited significantly larger increase with attention (*t*_31_ = 3.50, *p* = 0.001, *d* = 0.62) while FEP did not (*p* = 0.67). There was also a trend-level interaction between group and time (*F*_1,55_ = 3.08, *p* = 0.09) with HC exhibiting a non-significant increase (baseline: 0.024 ± 0.01; follow-up: 0.030 ± 0.02) and FEP exhibiting a non-significant decrease (baseline: 0.025 ± 0.01; follow-up: 0.020 ± 0.02) over time. No additional interactions were significant (*p*'s > 0.1).

### Comparison with participants lost to follow-up

Compared to FEP participants who returned for follow-up testing, those who did not were younger (23.8 ± 5.0 *v.* 20.3 ± 3.0; *p* = 0.02). Groups did not differ in any other demographic variables (*p*'s > 0.4), symptoms (*p*'s > 0.4), community functioning (*p*'s > 0.1), or EAGBR power or ITPC during either task condition (*p*'s > 0.2).

### Correlation with clinical outcomes and medication dose

**Figure 3.**
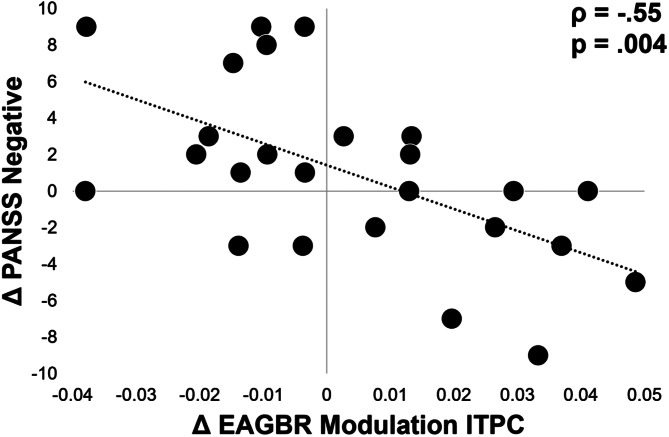
Correlation between increased EAGBR ITPC modulation and reductions in negative symptoms over time. PANSS, Positive and Negative Syndrome Scale.

There were no significant correlations between EAGBR modulation by attention and clinical assessments at either baseline or follow-up timepoints (*p*'s *>* 0.2). Among FEP, improved modulation of EAGBR ITPC by attention over time was associated with reductions in PANSS negative scores (*ρ* = −0.55, *q* = 0.09; Fig. 3). All uncorrected correlation statistics examining relationships with EAGBR modulation are presented in online Supplementary Table S1. No correlations between clinical ratings and EAGBR measures recorded during either condition survived correction for multiple comparisons. All uncorrected correlations with all EAGBR values are presented in online Supplementary Table S2.

Medication dose, converted into chlorpromazine equivalents, did not correlate with individual EAGBR values (*p*'s > 0.1) or their modulation by attention (*p*'s > 0.1) at either timepoint. To assess the potential influence of change in medication status on the relationship between improved ITPC and negative symptoms, correlations between these measures were conducted after removing the five participants with medication status change (four discontinued and one started medication). The relationship between ITPC and symptom improvement was slightly more robust (*ρ* = −0.60 *v.* −0.55) after removing these individuals.

## Discussion

Attentional control over sensory processing, exerted via coordinated inter-regional connectivity in the service of prioritizing environmental stimuli, reflects a critical executive function that appears to be disrupted in psychosis. The current investigation establishes the presence of this deficit proximal to disease onset and suggests that its recovery during an early stage of the illness is associated with symptom improvement. FEP were unable to enhance the EAGBR, a synchronization of high-frequency neural activity reflecting initial stage of cortical sensory processing, via attention. While this impairment persisted at follow-up testing at the group level, improvement in EAGBR modulation was associated with reductions in negative symptoms among FEP individuals. Thus, within the context of impaired attentional control of sensory gain, a subset of FEP that exhibited improved executive control experienced improved negative symptoms. Consistent with recent findings, the inability to modulate this response was observed despite an intact EAGBR among FEP, though a trend toward a diminished signal over time was present. These results support the growing literature elucidating the mechanisms of impaired executive control over the flow of information and their contribution to disease morbidity in early psychosis.

Impaired attentional modulation of the EAGBR mimics a similar deficit in the modulation of the downstream M100-related response previously observed in an overlapping FEP sample (Coffman et al., 2023; Curtis et al., 2023a). While impaired modulation of ITPC, but not power, suggests a greater susceptibility of neural synchrony in psychosis, EAGBR power may also be less sensitive to the effects of attention overall given the lack of a main effect of task condition and only a trend toward enhancement among HC (*p* = 0.08). This finding is discordant with previous studies identifying significant enhancement of EAGBR power with attention (Tiitinen et al., 1993; Debener et al., 2003), though this discrepancy may result from differences in methodologies between previous and current research, namely scalp electroencephalogram *v.* source-localized MEG recordings.

Contrary to the previous M100 results (Coffman et al., 2023), EAGBR modulation did not exhibit significant recovery at follow-up testing at the group level. However, improved modulation of this early sensory signal was associated with reductions in negative symptoms as measured by the PANSS, suggesting a potential role for EAGBR modulation in tracking disease progression during early stages of psychosis. Similar relationships between improved sensory-perceptual modulation and symptom reduction have been observed within the first year of illness (Coffman et al., 2023; Sklar et al., 2023). Unfortunately, as previously noted (Galderisi et al., 2021), the PANSS represents an imperfect measure of negative symptoms relative to newer measures such as the Brief Negative Symptom Scale and the Clinical Assessment Interview for Negative Symptoms given its more limited assessment of the experiential components of this symptom cluster. Of note, however, a similar correlation between the change in EAGBR ITPC modulation and negative symptoms over time was observed (*ρ* = −0.53, *p* = 0.007) using a PANSS scoring guideline recommended by the European Psychiatric Association to isolate core features of negative symptoms (Galderisi et al., 2021).

Despite an inability to enhance with attention, the EAGBR itself appears to be intact at this very early stage of psychotic illness. This attribute of the EAGBR provides an important distinction from previous work examining M100-response modulation, isolating a disruption in executive control from the potential confound of impaired sensory processing. An intact EAGBR proximal to disease onset is consistent with previous literature (Sklar et al., 2024; Spencer, Niznikiewicz, Shenton, & McCarley, 2008), though blunted EAGBRs have also been reported in psychosis (Leicht et al., 2010; Roach & Mathalon, 2008; Taylor et al., 2013). This discrepancy may be related to illness stage considering a recent longitudinal analysis revealing a preserved EAGBR at illness onset with deficits emerging 1 year later (Oribe et al., 2019). Interestingly, given previously documented relationships between larger EAGBRs and worse symptoms in early psychosis (Sklar et al., 2024; Taylor et al., 2013), the relative normalization of the EAGBR over time in this population may reflect a more appropriate excitatory inhibitory balance, a proposed neurochemical mechanism believed to underlie gamma-band response synchronization (Cardin et al., 2009; Sohal, Zhang, Yizhar, & Deisseroth, 2009). However, only a trend-level interaction between group and time, driven by non-significant changes within groups, was observed for EAGBR measures in the current study, possibly related to the shorter follow-up time window (4–12 months). Furthermore, while correlations between larger EAGBRs and worse negative symptoms as well as between EAGBR reductions and improved negative symptoms over time were observed (online Supplementary Table S2), these findings were exploratory and unlikely to survive correction for multiple comparisons.

### Limitations

Although dropout rates are comparable to other longitudinal first-episode and early psychosis studies, the loss of ~40% of FEP raises concerns for potential selection bias arising from differences in follow-up/drop-out groups. Fortunately, no discernible demographic, neurophysiological, or symptom-based differences were present between the groups at baseline. In general, the relatively smaller longitudinal sample size may have also led to increased risk of type II error with certain expected effects (i.e. attentional modulation of EAGBR power and correlations between larger EAGBR and worse symptoms) only achieving trend-level significance or failing to survive correction for multiple comparisons. The relatively few FEP who remained unmedicated at the time of testing also made it difficult to assess the impact of medications on outcome measures. However, medication dose was not associated with EAGBR measures and the relationship between changes in EAGBR modulation and symptoms over time persists following exclusion of FEP with medication status changes between the timepoints. In addition, as previously mentioned, the PANSS offers a more limited assessment of separable components of negative symptoms relative to second-generation assessment instruments and its reliance solely on behavioral observation undervalues the experiential aspect of patient symptoms. While use of an alternative scoring method using PANSS data meant to better capture negative symptom profiles yielded near-identical results, use of the Brief Negative Symptom Scale or Clinical Assessment Interview for Negative Symptoms would be warranted in future studies. Finally, the unbalanced racial distribution between the groups and its potential contribution to observed results cannot be overlooked.

## Conclusions

Individuals during early stages of psychosis exhibit a failure of executive control over the flow of information at the earliest stages of sensory processing within the cortex. In contrast to previous work, this deficit was observed despite an intact measure of sensory registration, mitigating potential confounds from faulty sensory processing. Recovery of sensory gain modulation over time appears to be associated with reductions in negative symptoms, elucidating a potential target for treatment of debilitating symptoms with limited therapeutic options in psychosis. Future studies should accelerate attempts to characterize disruptions in networks underlying impaired attentional modulation to facilitate future interventional approaches. Further work is also needed to clarify the paradoxical relationship between the EAGBR and symptoms which may reflect hyperexcitability of AC due to imbalance of neural excitation inhibition.

## Supporting information

Sklar et al. supplementary material Sklar et al. supplementary material

## Supplementary material

The supplementary material for this article can be found at https://doi.org/10.1017/S0033291724003052
