# Supplementary material for: Longitudinal evaluation of the early auditory gamma-band response and its modulation by attention in first-episode psychosis
Source: Psychol Med. 2024 Dec 2;54(15):4447–55. doi: 10.1017/S0033291724003052 (PMC11650157; doi:10.1017/S0033291724003052)
Supplement: Sklar et al. supplementary material [file S0033291724003052sup001.docx]

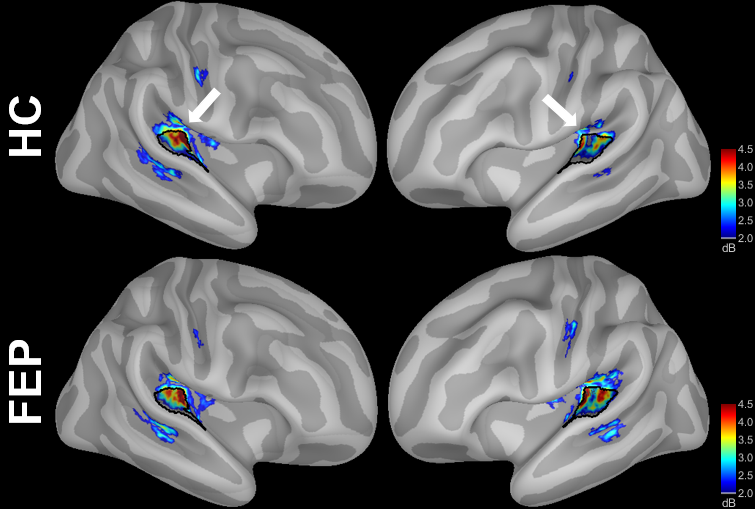


Supplemental Figure 1. Localization of EAGBR power, averaged across attention condition and time point, for healthy controls (HC) and first-episode psychosis (FEP) groups. While arrows indicate the location of our auditory cortex parcel (shaded in black).

|  | **PANSS Pos^a^** | **PANSS Neg^b^** | **GF:R^c^** | **GF:S^d^** |
| --- | --- | --- | --- | --- |
| **Baseline**  **Power Mod**  **ITPC Mod** | *ρ* = .04  *ρ =* .01 | *ρ =* -.20  *ρ =* -.26 | *ρ =* .15  *ρ =* .06 | *ρ =* .10  *ρ = .02* |
| **Follow-Up**  **Power Mod**  **ITPC Mod** | *ρ* = .11  *ρ =* .13 | *ρ =* -.12  *ρ =* -.09 | *ρ =* -.26  *ρ =* -.15 | *ρ =* -.01  *ρ =* -.05 |
| **Delta**  **Power Mod**  **ITPC Mod** | *ρ =* -.20  *ρ =* .00 | *ρ =* -.43*  *ρ =* -.55* | *ρ =* .25  *ρ =* .06 | *ρ =* .21  *ρ =* .16 |

Supplemental Table 1. Correlations between EAGBR modulation and clinical ratings

^a^Positive symptom component of the Positive and Negative Syndrome Scale; ^b^Negative symptom component of the Positive and Negative Syndrome Scale; ^c^Global Functioning: Role scale; ^d^Global Functioning: Social scale

**p*<.05

Supplemental Table 2. Exploratory correlations between EAGBR and clinical measures

|  | **PANSS Pos^a^** | **PANSS Neg^b^** | **GF:R^c^** | **GF:S^d^** |
| --- | --- | --- | --- | --- |
| **Baseline**  **Power Active**  **Power Passive**  **ITPC Active**  **ITPC Passive** | *ρ* = -.16  *ρ* = .08  *ρ =* .02  *ρ =* .07 | *ρ* = .20  *ρ* = .50*  *ρ =* .03  *ρ =* .18 | *ρ* = -.02  *ρ* = -.24  *ρ =* -.12  *ρ =* -.12 | *ρ* = .03  *ρ* = -.30  *ρ =* -.16  *ρ =* -.09 |
| **Follow-Up**  **Power Active**  **Power Passive**  **ITPC Active**  **ITPC Passive** | *ρ* = .10  *ρ* = -.02  *ρ =* .11  *ρ =* .03 | *ρ* = .27  *ρ* = .33  *ρ =* .23  *ρ =* .46* | *ρ* = -.26  *ρ* = -.07  *ρ =* -.23  *ρ =* -.12 | *ρ* = -.03  *ρ* = -.04  *ρ =* -.24  *ρ =* -.22 |
| **Delta**  **Power Active**  **Power Passive**  **ITPC Active**  **ITPC Passive** | *ρ* = .21  *ρ* = .02  *ρ =* -.05  *ρ =* -.04 | *ρ* = .15  *ρ* = .51*  *ρ =* .10  *ρ =* .45* | *ρ* = .11  *ρ* = -.16  *ρ =* .03  *ρ =* .01 | *ρ* = .25  *ρ* = -.07  *ρ =* .01  *ρ =* -.31 |

^a^Positive symptom component of the Positive and Negative Syndrome Scale; ^b^Negative symptom component of the Positive and Negative Syndrome Scale; ^c^Global Functioning: Role scale; ^d^Global Functioning: Social scale

**p*<.05
